# Covalent Organic Frameworks for Membrane Separation

**DOI:** 10.1002/advs.202412600

**Published:** 2024-12-11

**Authors:** Yuan‐Hang Jin, Meng‐Hao Li, Ying‐Wei Yang

**Affiliations:** ^1^ College of Chemistry Jilin University 2699 Qianjin Street Changchun 130012 P. R. China

**Keywords:** covalent organic frameworks, crystalline materials, functional materials, membrane separation, porous materials

## Abstract

Membranes with switchable wettability, solvent resistance, and toughness have emerged as promising materials for separation applications. However, challenges like limited mechanical strength, poor chemical stability, and structural defects during membrane fabrication hinder their widespread adoption. Covalent organic frameworks (COFs), crystalline materials constructed from organic molecules connected by covalent bonds, offer a promising solution due to their high porosity, stability, and customizable properties. The ordered structures and customizable functionality provide COFs with a lightweight framework, large surface area, and tunable pore sizes, which have attracted increasing attention for their applications in membrane separations. Recent research has extensively explored the preparation strategies of COF membranes and their applications in various separation processes. This review uniquely delves into the influence of various COF membrane fabrication techniques, including interfacial polymerization, layer‐by‐layer assembly, and in situ growth, on membrane thickness and performance. It comprehensively explores the design strategies and potential applications of these methods, with a particular focus on gas separation, oil/water separation, and organic solvent nanofiltration. Furthermore, future opportunities, challenges within this field, and potential directions for future development are proposed.

## Graphical abstract

The application of covalent organic frameworks (COFs) in membrane separation represents a significant breakthrough in materials science. This review explores the preparation strategies, synthesis methods, and application performance of COF membranes. It highlights the advantages of COFs in membrane separation and discusses the existing challenges and future development directions.

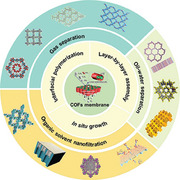

## 1 Introduction

Membranes are essential in energy and environmental fields as they facilitate selective separation processes vital for energy conversion,^[^ ^1^, ^2^, ^3^ ^]^ water treatment,^[^ ^4^, ^5^, ^6^ ^]^ and sensor detection.^[^ ^7^, ^8^, ^9^, ^10^ ^]^ Due to the eco‐friendliness, operational simplicity, and scalability, membrane separation has grown substantially in recent decades. By carefully designing membranes with specific pore sizes and functional groups, it is possible to selectively separate molecules or ions, enabling applications such as gas separation,^[^ ^11^, ^12^, ^13^, ^14^, ^15^, ^16^ ^]^ oil/water separation,^[^ ^4^, ^17^, ^18^, ^19^, ^20^, ^21^, ^22^, ^23^ ^]^ and organic solvent nanofiltration.^[^ ^24^, ^25^, ^26^, ^27^, ^28^, ^29^, ^30^, ^31^, ^32^ ^]^ A wide range of membranes have been developed, spanning pore sizes from micrometers to sub‐nanometers.^[^ ^33^ ^]^ These membranes are commonly made from amorphous polymers, including polyimide,^[^ ^34^, ^35^, ^36^, ^37^ ^]^ polyamide,^[^ ^38^, ^39^, ^40^ ^]^ polyacrylonitrile (PAN),^[^ ^41^, ^42^, ^43^, ^44^, ^45^ ^]^ and polyvinylidene fluoride.^[^ ^46^, ^47^, ^48^, ^49^, ^50^ ^]^ However, these polymers' disordered structure and low porosity can limit their molecular sieving efficiency. Membranes constructed from crystalline porous materials, such as zeolites and metal‐organic frameworks (MOFs), demonstrate markedly well‐defined pore structures and high porosity, resulting in superior selectivity and permeance.^[^ ^51^, ^52^, ^53^, ^54^, ^55^ ^]^ However, due to the high crystallinity of these materials, they exhibit weak mechanical strength and may crack during the membrane preparation process. Such cracking is commonly observed when materials are subjected to thermal shock following high‐temperature synthesis, during solvent removal via heating, or upon exposure to environmental contaminants.^[^ ^56^, ^57^ ^]^

Porous organic polymers, such as covalent organic frameworks (COFs), conjugated porous polymers, and porous aromatic polymers, offer significant advantages in separation applications due to their permanent porosity, large pore volumes, and exceptional structural stabilities.^[^ ^58^ ^]^ They have proven effective in various applications, such as carbon dioxide capture,^[^ ^59^ ^]^ iodine adsorption,^[^ ^60^ ^]^ dye adsorption,^[^ ^61^ ^]^ and water treatment.^[^ ^62^ ^]^ Among the numerous porous organic polymers, COFs have attracted extensive attention due to their structure‐property designability and customizable functions. COFs, a class of crystalline porous materials, are synthesized by linking organic units via covalent bonds.^[^ ^63^ ^]^ Their precise molecular arrangement enables the accurate placement of functional groups, resulting in customizable physical and chemical properties. COFs offer several advantages, such as exceptional structural regularity, high porosity, large surface areas, tunable structures, and an eco‐friendly nature. These properties make COFs ideal candidates for various applications, including gas storage,^[^ ^64^, ^65^, ^66^ ^]^ catalysis,^[^ ^67^, ^68^, ^69^, ^70^, ^71^, ^72^, ^73^ ^]^ drug delivery,^[^ ^74^, ^75^, ^76^, ^77^, ^78^ ^]^ and sensing.^[^ ^79^, ^80^, ^81^, ^82^ ^]^ The size, symmetry, and interconnection of organic linkers determine the configuration of COFs, allowing for precise control of pore size and facilitating the separation of molecules based on their sizes. The combination of these properties has made COFs a focal point of research in separation and a valuable material for membrane applications.

COF membranes have gained significant attention recently due to their potential for various separation applications. Unlike other thorough reviews that extensively detail the preparation methods and diverse applications of COF membranes from a comprehensive perspective, this review focuses on the latest advancements in COF membrane preparation, specifically highlighting interfacial polymerization, layer‐by‐layer assembly, and in situ growth. Particular emphasis is placed on how these methods influence the thickness and properties of the membranes. We also analyze membrane design strategies and evaluate the most suitable preparation methods for gas separation, oil/water separation, and organic solvent nanofiltration. A detailed introduction to these applications of COF membranes is provided, considering the underlying transport mechanisms: thermodynamic processes, kinetic processes, and molecular sieving. Finally, this review summarizes the opportunities and challenges faced by COF membranes in future practical applications and proposes directions for future development, offering a valuable reference for continued progress in this field.

## 2 Preparation Methods of COF Membranes

### 2.1 Design Principles

The design of COFs, particularly for membrane applications, necessitates careful consideration of both the inherent porosity and the selection of appropriate linkages to optimize functional properties. The two most common linkages for COF membranes are boronate ester and imine linkages. While boronate ester linkages provide COF membranes with high crystallinity and adjustable pore structures, their inherent instability in water due to susceptibility to hydrolysis poses a significant limitation.^[^ ^83^, ^84^ ^]^ In contrast, imine‐linked COFs exhibit greater chemical stability, remaining stable even in harsher environments.^[^ ^85^, ^86^ ^]^ The pore size of COF membranes, primarily determined by the size and shape of the constituent monomers, plays a crucial role in their separation performance. By carefully selecting monomers with specific geometries such as hexagons, triangles, or squares, precise control over pore aperture can be achieved.^[^ ^87^ ^]^ Furthermore, incorporating hydrophilic or hydrophobic groups during the synthesis process, known as functionalization, is a critical design parameter for modulating the selectivity and permeance of COF membranes.

The synthesis of COF membranes can be strategically tailored by selecting appropriate fabrication techniques based on the intrinsic properties of the COF monomers. Interfacial polymerization, which leverages the differential solubility of monomers in immiscible solvents, is particularly effective for creating thin, uniform COF membranes with controlled thickness and enhanced crystallinity.^[^ ^88^ ^]^ The layer‐by‐layer assembly method enables precise control over the thickness and composition of membranes by depositing COF dispersion. This adaptable technique can produce COF membranes with multiple functions and large areas.^[^ ^89^, ^90^ ^]^ In situ growth involves the direct polymerization of COF monomers on a substrate, ensuring strong adhesion and continuity between the substrate and the COF layer. This approach is practical for producing defect‐free membranes with robust mechanical properties.^[^ ^91^, ^92^ ^]^ By carefully selecting monomers and optimizing synthesis conditions, these fabrication strategies can effectively engineer COF membranes with desirable structural and functional properties for various separation applications. This section provides a detailed discussion of the three methods for preparing COF membranes suitable for separation: interfacial polymerization, layer‐by‐layer assembly, and in situ growth.

### 2.2 Interfacial Polymerization

Interfacial polymerization is the primary method for fabricating membranes, particularly for nanofiltration and reverse osmosis applications.^[^ ^93^ ^]^ This process involves polymerizing monomers at the interface of two immiscible phases, e.g., liquid‐liquid, vapor‐liquid, and solid‐vapor interfaces.^[^ ^94^ ^]^ During the interfacial polymerization process, the reaction occurs at the two‐phase interface, which is critical for controlling COF structure and uniform growth. The monomers are dissolved in the respective aqueous and organic phases. The reaction occurs at the interface between these two phases, where the diffusion and reaction kinetics of the monomers significantly influence the uniformity of the resulting COF membrane. The amine‐based monomer is typically dissolved in the aqueous phase, while the aldehyde or boronic acid monomer is dissolved in the organic phase.^[^ ^88^ ^]^ To enhance mechanical stability, COF membranes are often supported on substrates. By introducing functional groups onto the substrate, stronger interfacial interactions between the COF and the substrate can be achieved, resulting in improved structural integrity of the composite membrane.

**Figure 1:**
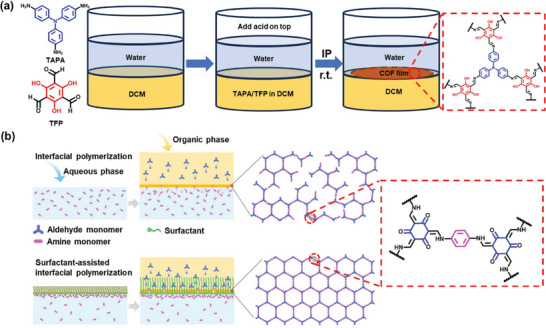
a) The process of creating self‐standing TAPA‐TFP COF membranes. Reproduced with permission.^[^ ^88^ ^]^ Copyright 2020, American Association for the Advancement of Science. b) Schematic illustration of COF membranes prepared by interfacial polymerization and surfactant‐assisted interfacial polymerization methods. Reproduced with permission.^[^ ^96^ ^]^ Copyright 2024, Elsevier.

Compared to traditional high‐temperature and high‐pressure methods for synthesizing COFs, interfacial polymerization offers a more practical approach for producing free‐standing COF membranes at room temperature, paving the way for broader application development. Liu, Han, Chung, and co‐workers successfully prepared flexible free‐standing COF membranes with molecular sieving capabilities using a biphasic liquid‐liquid interface method.^[^ ^88^ ^]^ As illustrated in **Figure** **1**a, tris(4‐aminophenyl)amine (TAPA) and 2,4,6‐triformylphloroglucinol (TFP) ligands were dissolved in dichloromethane, followed by the addition of an aqueous layer to form a biphasic interface. Polymerization proceeded rapidly at the interface of biphasic solvent, with the nanoparticle size modulated by introducing acetic acid as a catalyst. Increasing the amount of acetic acid from 1.8 to 3.6 mmol reduced the nanoparticle size from 1 to 0.1 µm. Moreover, the addition of surfactants can accelerate the interfacial polymerization process. For instance, sodium dodecyl sulfate (SDS) acted as a molecular bridge, facilitating the rapid formation of the COF membrane by promoting the transfer of amine monomers across the liquid‐liquid interface through electrostatic interactions.^[^ ^95^ ^]^ As shown in Figure 1b, surfactant‐assisted interfacial polymerization involves a monomer pre‐assembly process facilitated by surfactant molecules self‐assembling at the interface, improving interphase transport and allowing for the complete topological growth of the COF structure.^[^ ^96^ ^]^ Certain surfactants like SDS, sodium laureth sulfate, and sodium *N*‐lauroylsarcosinate, demonstrated the versatility of surfactant‐assisted interfacial polymerization method, which is particularly beneficial for monomers that polymerize slowly at the interface via reducing polymerization time and enhancing polymerization efficiency.

**Figure 2:**
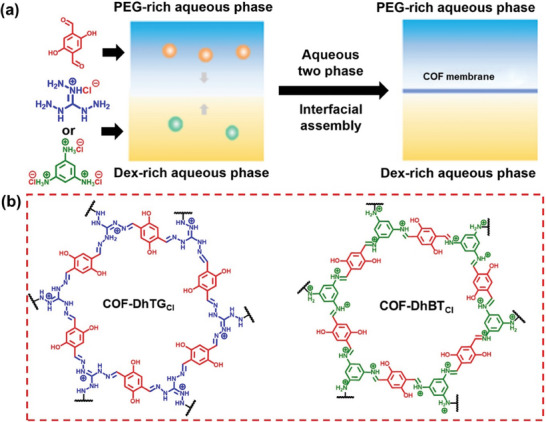
a) Aqueous two‐phase interfacial assembly of COF‐DhTG_Cl_ and COF‐DhBT_Cl_ membranes. b) Structure diagram of COF‐DhTG_Cl_ and COF‐DhBT_Cl_. Reproduced with permission.^[^ ^97^ ^]^ Copyright 2022, Springer.

In addition to the organic‐aqueous interface, interfacial polymerization can also occur at the interface between two aqueous phases. In 2022, Jiang, Pan, and co‐workers proposed the all‐aqueous system interface assembly method to produce COF membranes.^[^ ^97^ ^]^ The aqueous two‐phase system consisted of poly(ethylene glycol) (PEG) and dextran (Dex), which naturally separated into two immiscible aqueous phases with an interface region. Aldehyde (2,5‐dihydroxyterephthalaldehyde) and amine (triaminoguanidinium chloride and 1,3,5‐benzenetriamine trihydrochloride) monomers were distributed across the two aqueous phases, enabling the creation of COF‐DhTGCl and COF‐DhBTCl membranes at the interface through a 48–72 h polymerization process (**Figure** **2**). This solvent‐free approach serves as an environmentally friendly method for advanced membrane production.

**Figure 3:**
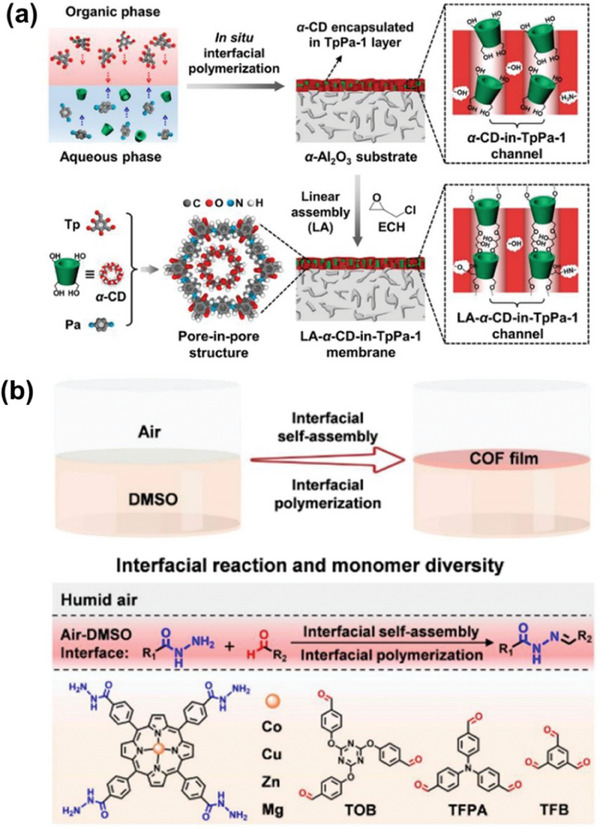
a) The method for synthesizing the LA‐*α*‐CD‐in‐TpPa‐1 membrane and the schematic presentation of pore‐in‐pore structures. Reproduced with permission.^[^ ^98^ ^]^ Copyright 2023, American Chemical Society. b) Process of preparing CoP‐TOB, CoP‐TFPA, and CoP‐TFB membranes utilizing vapor‐liquid interfacial polymerization and the reaction occurring between *meso*‐benzohydra‐zide‐substituted metal porphyrins and tris‐aldehydes (TOB, TFPA, and TFB). Reproduced with permission.^[^ ^99^ ^]^ Copyright 2023, Wiley VCH.

Interfacial polymerization often involves polymerizing a membrane on a specific substrate to facilitate membrane transfer and application. Caro and co‐workers employed *α*‐Al_2_O_3_ as a substrate at the junction of the organic and aqueous phase (**Figure** **3**a), successfully synthesizing a membrane (TpPa‐1) by the condensation of 1,3,5‐triformylphloroglucinol (Tp) and p‐phenylenediamine (Pa‐1) monomers.^[^ ^98^ ^]^ During the interfacial polymerization process, *α*‐cyclodextrin (*α*‐CD) molecules were embedded within the growing TpPa‐1 layer, resulting in their encapsulation within the structured 1D nanochannels of the membrane. Developing large‐area, self‐supporting membranes is essential for advancing membrane technology and continues to pose a significant challenge. Vapor‐liquid interfacial polymerization has shown promise in effectively addressing this issue. Fang, Cao, Zhao, and co‐workers fabricated free‐standing metalloporphyrin‐based COF membranes using vapor‐liquid interfacial polymerization. CoP‐TOB, CoP‐TFPA, and CoP‐TFB membranes were formed at the vapor‐liquid interface via the creation of acylhydrazone bonds, facilitated by the interaction of metal porphyrins substituted with *meso*‐benzohydrazide and tris‐aldehyde linkers (TOB, TFPA, and TFB) (Figure 3b). These membranes, with areas reaching up to 3000 cm^2^, displayed high mechanical stability, structural integrity, and hydrolytic stability due to the dynamic covalent acylhydrazone bonds.^[^ ^99^ ^]^ The ability to produce large‐area membranes with consistent properties is attributed to the precise control over COF membrane growth offered by vapor‐liquid interfacial polymerization.^[^ ^100^ ^]^ This method provides a simple and universal approach for preparing high‐quality COF membranes on a large scale.

**Figure 4:**
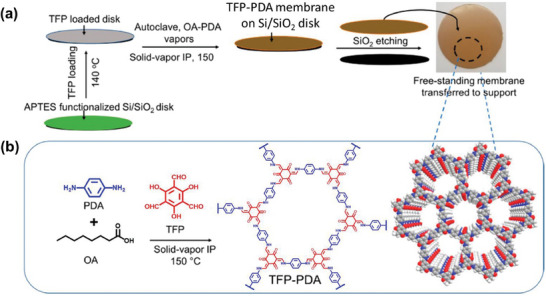
a) Schematic diagram of the fabrication process of the TFP‐PDA COF membrane through solid‐vapor interfacial polymerization. b) Structural diagram and digital photo of TFP‐PDA COF membrane. Reproduced with permission.^[^ ^101^ ^]^ Copyright 2020, American Chemical Society.

Solid‐vapor interfacial polymerization differs from previous methods by requiring a specific temperature and a more complex growth process for the COF membrane. In 2020, Jiang, Wu, and co‐workers successfully prepared a TFP‐PDA COF membrane using solid‐vapor interfacial polymerization for the first time. As shown in **Figure** **4**, the TFP‐PDA COF membrane was formed through the reaction of 1,3,5‐triformylphloroglucinol (TFP) monomers, loaded onto a (3‐aminopropyl)triethoxysilane‐functionalized Si/SiO_2_ disk, with *p*‐phenylenediamine (PDA) vapor monomers at 150 °C in an autoclave. Notably, the solid‐vapor interface allows for an accelerated polymerization reaction rate due to the increased temperature while maintaining interface integrity, enabling simultaneous polymerization and crystallization within 9 h.^[^ ^101^ ^]^

In summary, the interfacial polymerization method, while straightforward, faces significant challenges in terms of chemical stability, scalability, and pore size control. The reversible nature of covalent bonds, such as imine and boronate ester linkages, limits the long‐term stability of COF membranes. To address this, the development of more stable linkages is crucial. Additionally, current synthesis methods are primarily limited to laboratory‐scale production, hindering the commercialization of COF membranes. Scaling up the synthesis process while maintaining membrane integrity and performance remains a significant challenge. Furthermore, precise control over pore size, essential for specific separation applications, is difficult to achieve in interfacial polymerization due to the complex reaction dynamics at the phase interface.

### 2.3 Layer‐by‐Layer Assembly

As previously discussed, interfacial polymerization is a simple and effective method for producing high‐quality, large‐scale membranes. Despite its advantages, the precise control of membrane thickness and other structural properties remains challenging. This is where the layer‐by‐layer assembly technique enters the discussion, presenting an alternative method that facilitates improved control over the thickness of the membrane. Unlike interfacial polymerization, which relies on interfacial reactions between monomers, layer‐by‐layer assembly involves the sequential deposition of thin layers onto a substrate, enabling precise control over membrane thickness and composition.

**Figure 5:**
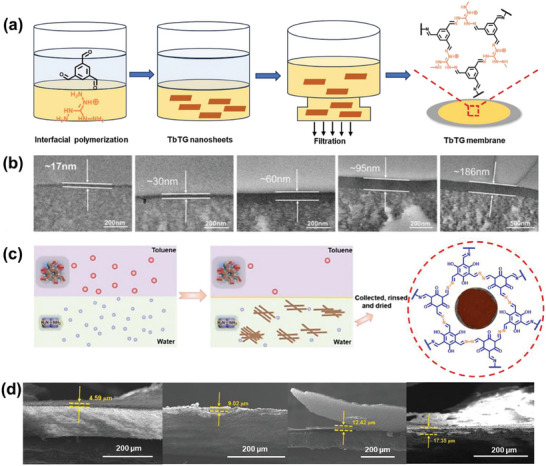
a) Depiction of the synthesis procedure for TbTG COF membranes and b) TEM images of the cross‐sections of membranes with varying thicknesses. Reproduced with permission.^[^ ^104^ ^]^ Copyright 2023, Elsevier. c) Schematic illustration of the formation process for the TpHZ COF. d) SEM images of cross‐sections of membranes with different thicknesses. Reproduced with permission.^[^ ^90^ ^]^ Copyright 2023, Elsevier.

Inspired by the success of single‐layer graphene and graphene oxide (GO) membranes, scientists have established a layer‐by‐layer stacking technique for producing COF membranes.^[^ ^102^, ^103^ ^]^ This method involves dispersing COF nanosheets in a solvent and then layering them onto a substrate to form a continuous membrane. The stacking process can be facilitated through vacuum‐assisted or dip‐coating methods. Due to their planar organic linkers, most COFs exhibit a 2D layered structure. By breaking down bulk COFs into 2D nanosheets, van der Waals interactions between neighboring layers can be interrupted, enabling the creation of continuous COF membranes through layer‐by‐layer assembly. This technique allows for precise control over membrane thickness, which is crucial for optimizing separation efficiency and permeance. While thicker membranes generally exhibit higher separation efficiency, they often have lower permeance. Therefore, membranes of varying thicknesses are fabricated to determine the optimal balance between these two factors. As shown in **Figure** **5**a, 2D TbTG (Tb: 1,3,5‐benzenetricarboxaldehyde; TG: triaminoguanidine chloride) nanosheets, measuring 1.5 nm in thickness, were prepared through liquid‐liquid interfacial polymerization. These nanosheets were then assembled onto a porous polyacrylonitrile substrate using vacuum‐assisted self‐assembly to create a TbTG COF membrane.^[^ ^104^ ^]^ The TbTG COF membrane exhibited sub‐nanometer pore size and demonstrated excellent molecular sieving ability. By systematically varying the volume of TbTG nanosheet dispersions from 25 to 400 µL, researchers could precisely control the membrane thickness, ranging from 17 to 186 nm (Figure 5b). Optimal membrane performance, balancing separation efficiency and permeance, was achieved when the TbTG COF membrane reached a thickness of 95 nm. In 2023, Liu and co‐workers successfully developed durable and loosely arranged TpHZ COF membranes by filtering TpHZ COF nanofibers. The TpHZ COF membrane exhibited exceptional super‐wettability, allowing for switchable transport performance. This property enables controlled separation of various oil‐water mixtures and emulsions, achieving high separation efficiency and permeance (Figure 5c). The thickness of the TpHZ COF membranes was precisely controlled by varying the amounts of TpHZ COF nanofibers during the filtration assembly process. Achieved thicknesses ranged from 0 to 17.35 µm, corresponding directly to TpHZ COF nanofiber loadings from 0 to 1.08 mg cm^−2^, resulting in a transition from hydrophobic to superhydrophilic properties (Figure 5d).^[^ ^90^ ^]^ Thicker TpHZ COF membranes exhibited improved underwater superoleophobic and underoil superhydrophobic characteristics due to their increased capacity to retain oil and water, leading to enhanced separation efficiency. Particularly, membranes with thicknesses of 12.42 and 17.35 µm demonstrated outstanding separation performance, achieving separation efficiency greater than 99% for different oil‐water mixtures. Consequently, in practical applications, the membrane thickness can be chosen according to the particular demands of the task. A thicker membrane is recommended if high separation efficiency is prioritized; conversely, a thinner membrane should be selected if high permeance is more critical.

**Figure 6:**
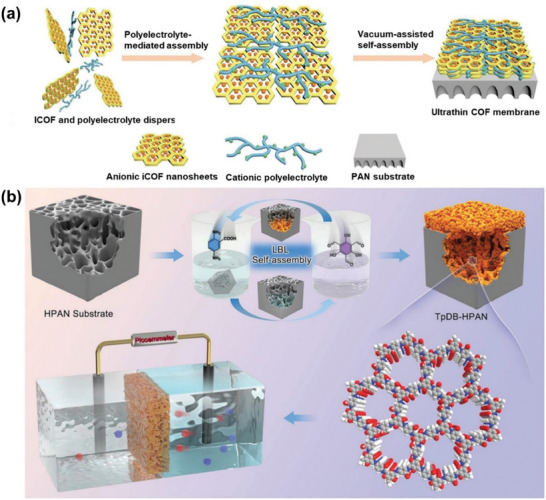
a) Schematic of polyelectrolyte‐mediated assembly strategy for the fabrication of ultrathin ionic COF membranes composed of TpPa‐SO_3_H. Reproduced with permission.^[^ ^105^ ^]^ Copyright 2023, Wiley‐VCH. b) Schematic diagram of the synthesis of TpDB‐HPAN COFs membranes through layer‐by‐layer (LBL) self‐assembly at ambient temperature, TpDB structure, and the salinity gradient energy conversion process. Reproduced with permission.^[^ ^106^ ^]^ Copyright 2023, Wiley‐VCH.

For specific types of COFs, such as ionic COFs, researchers can employ a polyelectrolyte‐mediated assembly strategy (**Figure** **6**a). First, 1,3,5‐trihydroxybenzene (Tp) and 2,5‐phenylenediamine disulfonate (Pa‐SO_3_H) were reacted under acidic conditions at 120 °C for 72 h to obtain TpPa‐SO_3_H. Then, employing this strategy effectively regulated the assembly modalities involving polyethyleneimine (PEI) and TpPa‐SO_3_H, leading to the formation of ultra‐large ionic COF nanosheets bridged by PEI. These nanosheets can be subsequently transformed into membranes of TpPa‐SO_3_H ionic COFs, achieving a thickness of merely 8 nm. As membrane thickness decreases, mass transfer resistance is significantly reduced, leading to increased gas permeance. This method is effective for producing thinner separation membranes.^[^ ^105^ ^]^ The TpDB‐HPAN COF membrane was also successfully employed for energy harvesting, fabricated through a layer‐by‐layer self‐assembly method at ambient temperature (Figure 6b).^[^ ^106^ ^]^ The synthesis of the carboxyl‐rich TpDB COF involved the assembly of Tp and 2,5‐diaminobenzoic acid (DB) monomers on the substrate. The resulting TpDB‐HPAN membranes exhibited an enhanced open‐circuit voltage (*V*_oc_), leading to remarkable energy harvesting performance.

The layer‐by‐layer method offers promising prospects for preparing COF membranes, as it allows for precise control over membrane thickness. The scalability of this method, particularly for producing COF membranes with larger surface areas, is a crucial area for future development. However, the durability of membranes prepared using the layer‐by‐layer technique remains a significant challenge. Research should focus on enhancing the chemical durability of these membranes by incorporating functional groups to broaden their application in separation processes.

### 2.4 In Situ Growth

**Figure 7:**
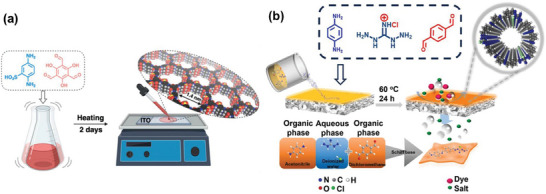
a) Schematic illustration of the manufacturing procedure of the TpPa‐SO_3_H membrane. Reproduced with permission.^[^ ^92^ ^]^ Copyright 2024, American Chemical Society. b) The process of preparing ionic COF composite membranes. Reproduced with permission.^[^ ^107^ ^]^ Copyright 2024, Elsevier.

In situ growth is a widely recognized technique for synthesizing COF membranes, where the membranes are grown directly on a suitable substrate to achieve a continuous, uniform, and highly crystalline structure. Continuous COF membranes are recognized for their superior separation performance. In 2024, Khashab and co‐workers successfully synthesized a TpPa‐SO_3_H membrane using in situ growth on an indium tin oxide (ITO)‐coated glass substrate (**Figure** **7**a). Initially, Tp (10 mM) and Pa‐SO_3_H (13 mM) were dissolved in N‐methylpyrrolidone and dimethyl sulfoxide, followed by ultrasonic treatment. The resulting solution was subsequently applied to the ITO glass surface and allowed to react at 60 °C for 48 h, leading to the formation of a continuous TpPa‐SO_3_H membrane.^[^ ^92^ ^]^ The TpPa‐SO_3_H membrane demonstrated notable crystallinity and featured vertically aligned 1D nanochannels functionalized with sulfonic acid groups. The addition of anionic sulfonic acid components significantly improved the membrane's properties. To address the common issue of insufficient mechanical strength in self‐supporting COF membranes, researchers have explored in situ COF membrane growth on non‐woven plates as an alternative approach. Cao and co‐workers developed a novel membrane fabrication technique, referred to as the altered casting‐precipitation‐evaporation method. This method involved preparing a substrate on non‐woven plates and then immersing them in a monomer solution for in situ COF membrane growth (Figure 7b).^[^ ^107^ ^]^ This process began by casting a polyacrylonitrile solution, polyvinylpyrrolidone, and dimethylformamide onto a non‐woven plate to form a substrate membrane. This substrate was then coagulated in deionized water. The hydrolysis of the substrate membrane occurred in a sodium hydroxide solution, resulting in hydrolyzed PAN, which acted as the support layer. The ionic COF layer was synthesized directly on the hydrolyzed PAN substrate via a Schiff base reaction involving p‐phenylenediamine and p‐phthalaldehyde monomers, resulting in the formation of the composite membrane.

The in situ growth method is a significant approach for preparing COF membranes, particularly in the development of COF membranes with specific separation properties. Future trends in in situ growth may involve selecting substrates with diverse functionalities, allowing for the customization of COF membranes to meet various application requirements. However, this method also encounters chemical stability challenges similar to those of other methods, necessitating the development of robust linkages COF structures with enhanced stability to address this issue.

## 3 Separation Applications of COF Membranes

**Figure 8:**
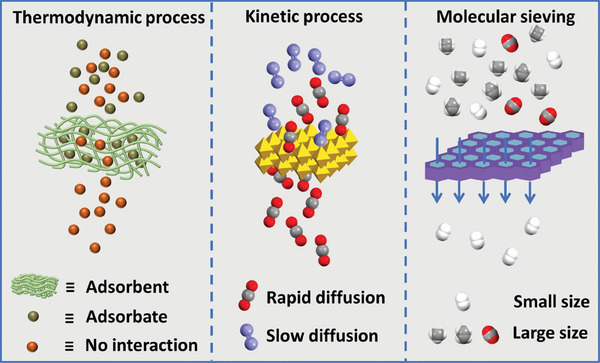
The transport mechanism diagram of the thermodynamic process, kinetic process, and molecular sieving.

From a transport mechanism perspective, the separation process primarily involves three methods: (1) Thermodynamic process, where material adsorption leads to thermodynamic equilibrium between the adsorbate and adsorbent, resulting in separation. (2) Kinetic process, where separation occurs due to the varying diffusion rates of components within the mixture. (3) Molecular sieving, where separation is achieved based on the size or shape differences of the mixture components (**Figure** **8**). Previous studies have employed common separation materials, including supramolecular polymer,^[^ ^108^, ^109^, ^110^, ^111^, ^112^ ^]^ MOFs,^[^ ^113^, ^114^, ^115^ ^]^ and COFs,^[^ ^24^, ^116^, ^117^, ^118^, ^119^ ^]^ to separate substances using the above three main methods. In 2019, we introduced a fluorescent supramolecular polymer capable of both detecting and removing mercury(II) ions. The polymer, formed via host‐guest interactions between a functionalized pillar[6]arene and a tetraphenylethylene‐based guest, displays aggregation‐induced emission when it binds with Hg^2+^. This system demonstrates exceptional selectivity, sensitivity, and recyclability in detecting and eliminating mercury from water, making it a promising candidate for environmental cleanup applications.^[^ ^111^ ^]^ Liu and co‐workers innovatively developed highly oriented UiO‐66 membranes with a thickness of 165 nm. They began by synthesizing uniform triangular UiO‐66 nanosheets, ≈40 nm thick, using a unique anisotropic etching method. Subsequently, these nanosheets were subjected to confined counter‐diffusion‐assisted epitaxial growth to create the oriented membranes.^[^ ^120^ ^]^ This breakthrough resulted in a notable reduction in the membrane's thickness and the diffusion barrier within the framework, achieving remarkable CO_2_ permeance of 2070 GPU along with a CO_2_/N_2_ selectivity of 35.4. Jiang and co‐workers optimized the effectiveness of COF membranes for organic solvent nanofiltration by tailoring the pore structure.^[^ ^121^ ^]^ They integrated COF layers composed of TAPB‐TPOC_x_ monomers with different alkyl side chain lengths (x = 1, 4, 6, 8) onto polyacrylonitrile substrates, allowing precise control over pore sizes and chemical environment within the COF selective layer. The resulting membranes exhibited tunable molecular weight cut‐offs, ranging from 330 to 970 g mol^−1^, making them suitable for a wide range of organic solvent separations.

To optimize the separation efficiency of COF membranes, a synergistic approach that integrates thermodynamics, kinetics, and molecular sieving mechanisms is essential. COF membranes can achieve efficient separations through surface functionalization for selective adsorption, tunable pore structures for tailored diffusion rates, and precise pore size control for molecular sieving. By incorporating specific functional groups, adsorption selectivity can be enhanced through increased interactions with target molecules, promoting selective thermodynamic equilibrium. For kinetic control, pore size engineering can be employed to modulate diffusion rates and accelerate the separation of target species. Simultaneously, maintaining pore size uniformity and framework rigidity is critical for effective molecular sieving, where solutes are selectively excluded based on size and shape.

### 3.1 Gas Separation

**Figure 9:**
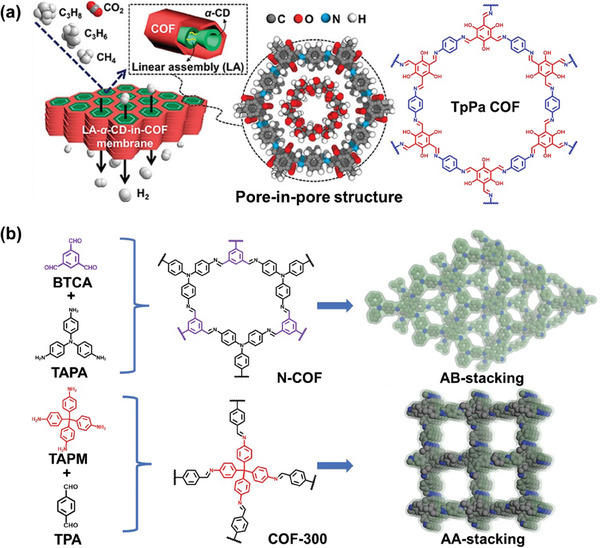
a) Schematic diagram of the pore‐in‐pore structure of LA‐*α*‐CD‐in‐TpPa‐1 membrane and the process to achieve gas separation. Reproduced with permission.^[^ ^98^ ^]^ Copyright 2023, American Chemical Society. b) Structure and composition of N‐COF (top) membrane and COF‐300 (down) membrane. Reproduced with permission.^[^ ^122^ ^]^ Copyright 2023, Wiley‐VCH.

The selective gas separation of COF membranes relies on both kinetic and molecular sieving processes. Hydrogen is a clean energy alternative to fossil fuels, but its production also generates unwanted gases. Therefore, developing a COF membrane that can effectively separate hydrogen from mixed gases is crucial. Caro and co‐workers effectively created a COF membrane of LA‐*α*‐CD‐in‐TpPa‐1 through a pore‐in‐pore engineering technique.^[^ ^98^ ^]^ This innovative method involved reducing pore size by encapsulating *α*‐cyclodextrin (*α*‐CD) within the TpPa‐1 framework, thereby creating pathways for H_2_ transport and facilitating the selective separation of H_2_ from gases like C_3_H_8_, C_3_H_6_, and CH_4_ (**Figure** **9**a). The LA‐*α*‐CD‐in‐TpPa‐1 membrane exhibited a remarkable H_2_ permeance of ≈3000 GPU and showed improved H_2_ selectivity compared to CO_2_ and CH_4_, surpassing 30. The separation performance of the LA‐*α*‐CD‐in‐TpPa‐1 membrane for H_2_/CO_2_ and H_2_/CH_4_ surpassed the Robeson upper bounds, positioning it as one of the most efficient membranes for selective H_2_ separation. The pore‐in‐pore engineering strategy offers a novel concept for COF membranes to achieve gas separation through molecular sieving.

Adsorption is a strategy widely utilized for COF membrane separation. In the last five years, H_2_ permeance has reached 5160 GPU through the selective adsorption of other gases, achieving the highest reported H_2_ permeance in COF membranes.^[^ ^122^ ^]^ This study demonstrated that the two COF membranes can exhibit high H_2_/CO_2_ selectivity by utilizing the synergistic combination of adsorption and diffusion rather than relying solely on molecular sieving (Figure 9b). The imine‐based COF primarily adsorbs CO_2_, allowing H_2_ to flow smoothly through the pores. Adjusting the transmembrane pressure can influence the adsorption process. At high pressure, poor adsorption occurs as CO_2_ passes through the pores. When the pressure decreases, the membrane's ability to adsorb CO_2_ returns to normal. The fixed pore sizes of the membranes studied in this research promote H_2_ passage, while their self‐supporting structure helps to shield against external interference. Compared to COF membranes that rely exclusively on pore structure modifications for molecular sieving, these membranes demonstrate enhanced permeance and selectivity.^[^ ^123^ ^]^ Furthermore, this work explored the impact of stacking 2D COF nanosheets on separation performance. Compared to COF‐300, N‐COF possesses higher H_2_/CO_2_ selectivity but lower H_2_ permeance. This difference can be attributed to N‐COF's AB stacking structure, resulting in smaller pore sizes. Thus, AB stacking is an effective strategy for reducing pore size in COF membranes.

**Figure 10:**
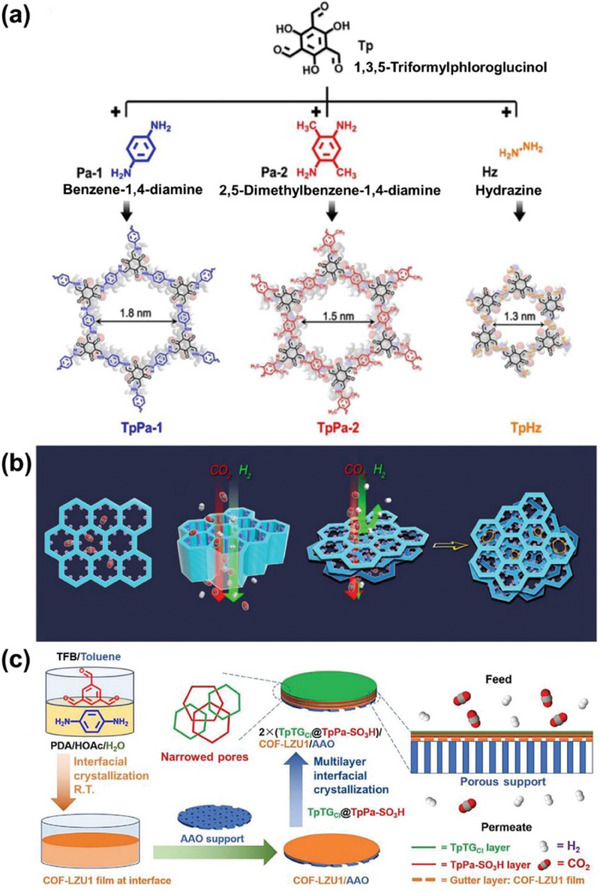
a) Structure and composition of TpPa‐1, TpPa‐2, and TpHZ. b) Schematic diagram of the process of separating CO_2_/H_2_ with TpPa‐2 nanosheets. Reproduced with permission.^[^ ^89^ ^]^ Copyright 2021, Wiley‐VCH. c) The method of multi‐interfacial engineering was implemented to develop ultrathin TpTG_Cl_ and TpPa‐SO_3_H COF membranes for both manufacturing and gas separation purposes. Reproduced with permission.^[^ ^124^ ^]^ Copyright 2021, Wiley‐VCH.

In 2021, Yang, Peng, and co‐workers synthesized three β‐ketoenamine‐based COF nanosheets (TpPa‐1, TpPa‐2, and TpHz) and assembled them into ultra‐thin membranes with a distinctive staggered stacking pattern, leading to successful CO_2_/H_2_ separation (**Figure** **10**a).^[^ ^89^ ^]^ To control the pore size of COF membranes, researchers utilized the hot‐drop coating (HDC) method instead of traditional solvothermal and interfacial polymerization techniques. This approach facilitated the staggered topology of COF nanosheets and promoted their close‐packing mode. This architectural design enhances CO_2_‐selective permeance through a combination of selective adsorption and surface diffusion. The TpPa‐1, TpPa‐2, and TpHz nanosheets preferred CO_2_ adsorption due to specific interactions at functionalized sites within the framework. This facilitated the diffusion of CO₂ across the membrane surface while effectively excluding smaller molecules like H₂ due to size‐selective effects (Figure 10b). Also, layer‐by‐layer interfacial reactions can lead to the formation of narrow pores at COF‐COF interfaces. Zhao and co‐workers employed a multi‐interface engineering strategy to synthesize two COFs with mismatched pore sizes (TpTG_Cl_ and TpPa‐SO_3_H), developing narrow pores at the COF‐COF interface.^[^ ^124^ ^]^ These COF layers were grown on the COF‐LZU1 membrane gutter layer, which features comparatively large pores, facilitating the formation of defect‐free membranes (Figure 10c). The resulting TpTG_Cl_@TpPa‐SO_3_H membrane exhibited an H_2_ permeance of 2163 GPU and an H_2_/CO_2_ selectivity of 26. This approach demonstrates that achieving COF narrow pores through diverse COF multi‐interface engineering can enhance H_2_ permeance and selectivity.^[^ ^125^ ^]^

**Figure 11:**
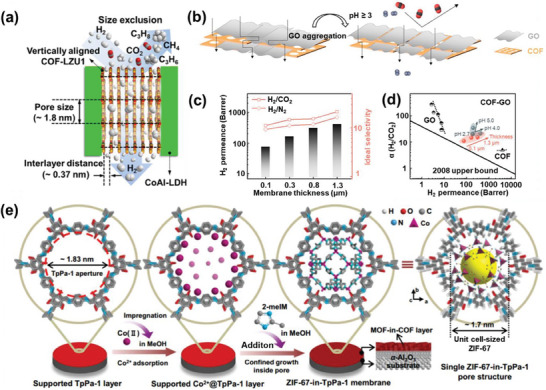
a) Schematic illustration of the gas permeance through a COF‐LZU1 membrane aligned vertically. Reproduced with permission.^[^ ^126^ ^]^ Copyright 2020, American Chemical Society. b) Schematic diagram of pH‐regulated self‐assembly of TAPB‐BTCA COF‐GO composite membranes with varying thickness to control gas selectivity and permeance. c) The H_2_ permeance and ideal selectivity (H_2_/CO_2_ and H_2_/N_2_) of TAPB‐BTCA COF‐GO membrane with different thickness. d) The gas separation performance of TAPB‐BTCA COF‐GO. Reproduced with permission.^[^ ^123^ ^]^ Copyright 2024, Elsevier. e) Schematic diagram of ZIF‐67‐in‐TpPa‐1 membrane synthesis and single pore structure. Reproduced with permission.^[^ ^127^ ^]^ Copyright 2021, Springer Nature.

Utilizing vertically oriented “templates” to facilitate the vertical growth of COF can effectively create narrow pores. In 2020, Caro, Fan, and co‐workers prepared hybrid membranes (COF‐LZU1) by in situ orientation on vertically aligned CoAl‐layered double hydroxide (LDH) nanosheets.^[^ ^126^ ^]^ The COF‐LZU1 membrane demonstrated an H_2_ permeance of 3600 GPU, achieving a selectivity of 31.6 for H₂/CO₂ and 29.5 for H_2_/CH_4_. The vertical COF‐LZU1 membrane's significant permeance can be attributed to the straight transport pathways established within the interlayer spaces (**Figure** **11**a), employing vertical CoAl‐LDH nanolayers as “templates” for vertically aligned 2D COF layers. This method overcomes the limitations associated with the parallel layered structure of traditional 2D COF membranes by innovatively utilizing the gaps between the COF layers as gas separation channels. Additionally, COF membranes with a planar layered structure are typically combined with other 2D materials for enhanced gas separation. Andreeva and co‐workers employed a pH‐assisted self‐assembly technique to improve the interactions of surface charges between COFs and GO nanosheets, resulting in membranes with improved permeance and selectivity (Figure 11b). The TAPB‐BTCA COF, synthesized using an environmentally friendly approach, was selected for its compatibility with water‐based processes. Through experimentation, it was determined that by modifying the COF/GO ratio and assembly pH, the membrane structure could be precisely adjusted, effectively addressing the conventional trade‐off between permeance and selectivity (Figure 11c,d).^[^ ^123^ ^]^ Beyond 2D materials, COFs can be integrated with 3D materials to form micro/nanopore networks, further enhancing separation efficiency.^[^ ^127^ ^]^ Meng, Caro, and co‐workers introduced a novel molecular sieving membrane utilizing MOF‐in‐COF technology for the highly selective separation of H_2_ (Figure 11e). By restricting the growth of cobalt‐based ZIF‐67 MOFs within the 2D TpPa‐1 COF layer, the researchers successfully developed a distinct network of micro/nanopores. This combined structure leverages the precise molecular sieving properties of ZIF‐67 MOFs with the rapid transport channels of TpPa‐1 COFs. The resulting ZIF‐67‐in‐TpPa‐1 membranes demonstrated exceptional H_2_ permeance (>3000 GPU) and marked enhancements in selectivity for H_2_ compared to other gases like CO_2_ and CH_4_, surpassing the Robeson upper bounds.^[^ ^128^ ^]^

### 3.2 Oil/Water Separation

The release of oily wastewater and oil spills has significantly contaminated the ecological environment. Developing effective membranes for oil/water separation is essential for protecting water resources. COFs, with their well‐defined pore structures and diverse functional groups, exhibit a remarkable ability to transition between hydrophilic and hydrophobic states. This unique property makes COF membranes highly suitable for oil/water separation.

**Figure 12:**
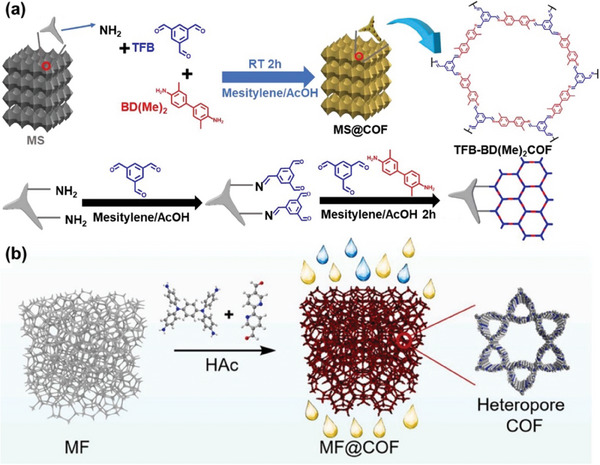
a) Schematic illustration of the fabrication process of MS@COF. Reproduced with permission.^[^ ^129^ ^]^ Copyright 2023, Elsevier. b) Preparation of hydrophobic MF@COF composite. Reproduced with permission.^[^ ^130^ ^]^ Copyright 2023, Elsevier.

The fabrication of COF membranes from powdered materials has consistently been a hurdle in the development of advanced oil/water separation technologies. Researchers have been exploring the deposition of COFs onto various substrates to create composite materials suitable for oil/water separation. Recent studies have employed in situ growth to decorate the COFs onto the surfaces of melamine sponge (MS@COF)^[^ ^129^ ^]^ and melamine foam (MF@COF)^[^ ^130^ ^]^ (**Figure** **12**a,b). COFs possess high specific surface areas, strong adsorption capacities, and excellent chemical stability, while melamine is characterized by a hierarchical pore structure, lightweight nature, and high adsorption capacity. Both MS@COF and MF@COF inherit the advantageous properties of COFs and melamine. The rate at which oil‐water mixtures are separated is sustained at 50 mL min^−1^ through the use of a peristaltic pump, while the oil adsorption capacity of MS@COF stands out at a remarkable 160 g g^−1^. Furthermore, MS@COF is proficient at separating stable water‐in‐oil emulsions, achieving an impressive separation efficiency of 98.8%. Even after undergoing 50 cycles of separation, the membranes made of MS@COF maintained their capacity to separate oil‐water mixtures and emulsions, showcasing excellent recyclability. MF@COF membranes exhibited desirable properties, including highly hierarchical pores, strong hydrophobicity, ultralight weight, robust stability, good mechanical elasticity, and processability, making them well‐suited for sample processing in non‐target food safety analysis and oil/water separation. In a mere 30 s, nearly complete removal of the matrix was achieved in 6 kinds of representative fat‐rich foods, with 323 chemical hazards being full recovered.

**Figure 13:**
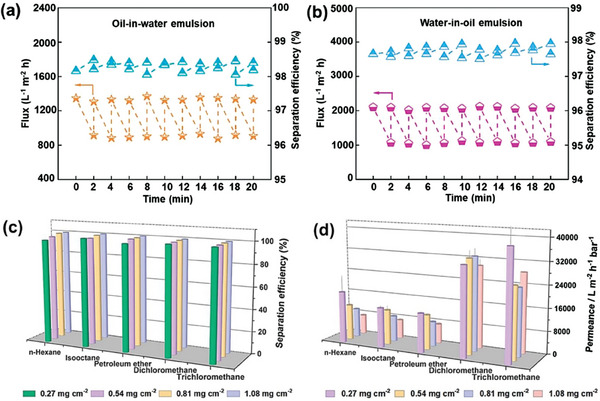
The performance of TPB‐DMTP‐COF/PAN membrane in separating oil‐in‐water emulsion a) and water‐in‐oil emulsion b). Reproduced with permission.^[^ ^131^ ^]^ Copyright 2023, Elsevier. The separation efficiency c) and permeance d) of the TpHZ COF membranes containing varying amounts of TpHZ COFs for different oil/water mixtures. Reproduced with permission.^[^ ^90^ ^]^ Copyright 2023, Elsevier.

The polymer substrates typically possess favorable mechanical properties, thermal stability, and processability. In a recent study, Zhang, Han, and co‐workers are the first to prepare imine‐linked COF nanospheres (TPB‐DMTP‐COF).^[^ ^131^ ^]^ TPB‐DMTP‐COF was suspended in water and used to functionalize a PAN substrate, resulting in a dual superlyophobic COF membrane (TPB‐DMTP‐COF/PAN). The membrane demonstrated significant separation fluxes and efficiencies for both oil‐in‐water and water‐in‐oil emulsions, exceeding 1200 L m^−2^ h^−1^ and 98.0%, and 2100 L m^−2^ h^−1^ and 97.4%, respectively (**Figure** **13**a,b). Furthermore, the ultra‐low adhesion in a liquid allowed the TPB‐DMTP‐COF/PAN membrane to exhibit excellent reusability and antifouling properties. The PAN substrate exhibits excellent chemical and thermal stability; other substrates, such as polyvinylidene fluoride, can also achieve efficient oil/water separation when loaded with COFs. TpHZ COF nanofibers can be effectively loaded onto a polyvinylidene fluoride substrate through vacuum‐assisted assembly to create superhydrophobic or superoleophobic TpHZ COF membranes, either under oil or underwater.^[^ ^90^ ^]^ All prepared TpHZ COF membranes demonstrated separation efficiencies exceeding 99.0% for oil/water mixtures (Figure 13c). At low loading, the highest permeance was observed when separating a trichloromethane/water mixture, reaching 38 842 L m^−2^ h^−1^ bar^−1^ (Figure 13d). Considering separation efficiency and permeance, the TpHZ COF membrane with a thickness of 12.42 µm (0.81 mg cm^−2^) exhibited optimal separation performance.

**Figure 14:**
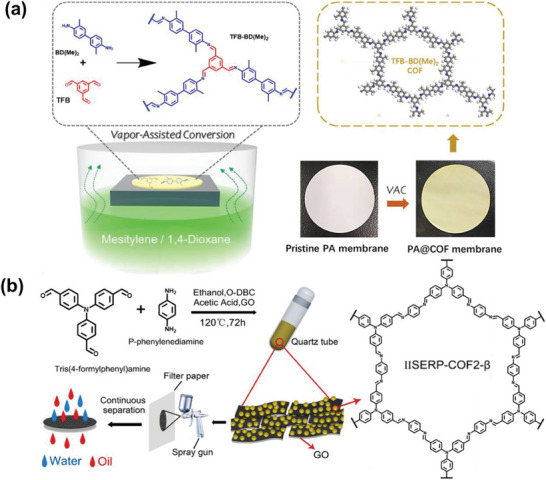
a) Scheme of the vapor‐assisted conversion setup and the topological structure of TFB‐BD(Me)_2_ COF. Reproduced with permission.^[^ ^132^ ^]^ Copyright 2022, Royal Society of Chemistry. b) Schematic illustration of the one‐step fabrication method for COF/GOx composites, followed by the coating process, which involves spraying the composite onto filter paper to facilitate oil/water separation. Reproduced with permission.^[^ ^133^ ^]^ Copyright 2021, Elsevier.

Other polymers, such as polyamide, can serve as the substrates for TFB‐BD(Me)_2_ COF, enabling the fabrication of TFB‐BD(Me)_2_ COF‐coated polyamide membranes via vapor‐assisted conversion (**Figure** **14**a). The TFB‐BD(Me)_2_ COF membrane showed notable hydrophobic properties, with a water contact angle of ≈100°, a stark contrast to the superhydrophilic nature of the unmodified polyamide membrane. This altered membrane exhibited remarkable separation capabilities for various water‐in‐oil emulsions, achieving a separation efficiency of up to 99.7% and a flux of 4583 L m^−2^ h^−1^ under modest negative pressure conditions. Moreover, both the separation efficiency and flux remained consistent throughout ten cycles of application to a water‐in‐o‐dichlorobenzene emulsion, demonstrating its strong reusability.^[^ ^132^ ^]^ Additionally, inorganic materials can serve as substrates for COFs, enhancing separation efficiency. The incorporation of GO nanosheets as substrates for COFs allows for improved membrane separation performance through the synergistic interaction between COFs and GO nanosheets. In 2021, Men, Zhang, Singh, and co‐workers formulated a simple one‐step technique to create hybrid materials that include IISERP‐COF2‐β along with GO.^[^ ^133^ ^]^ The IISERP‐COF2‐β particles were grown on GO sheets via covalent linking through a Schiff base interaction involving p‐phenylenediamine and tris(4‐formylphenyl)amine. The resulting IISERP‐COF2‐β/GO membrane exhibited synergistic effects. IISERP‐COF2‐β enhanced the acid/base resistance of GO, while GO mitigated the agglomeration of IISERP‐COF2‐β particles. The IISERP‐COF2‐β/GO, without any further surface modifications, were spray‐coated onto filter paper to form superhydrophobic coatings (Figure 14b). These coatings exhibited exceptional oil/water separation fluxes reaching as high as 26 960 L m^−2^ h^−1^ bar^−1^, while the separation efficiencies surpassed 98.0% for various oil/water mixtures. As COF membranes advance and separation demands diversify, a growing range of materials are employed as COF substrates. For instance, microchannels within wood can facilitate the in situ growth of TAPB‐PDA COF, which is utilized for the treatment of wastewater and the separation of oil/water.^[^ ^134^ ^]^ The smooth surface and fiber structure of cotton fabrics can also be employed to attach TFB‐BD(Me)_2_ COF for effective water‐oil emulsion separation.^[^ ^135^ ^]^ Moreover, the robust mechanical strength of stainless steel enables the coating of BTADHBD COF onto stainless steel mesh, promoting effective oil/water separation in harsh environments.^[^ ^136^ ^]^ Thus, the selection of substrates can be tailored to meet specific separation applications under a variety of challenging conditions.

### 3.3 Organic Solvent Nanofiltration

Organic solvent nanofiltration (OSN) is a membrane‐based technology with pore sizes ranging from 1 to 2 nm. This method is specifically designed to efficiently separate, purify, and recycle organic solvents. Beyond solvent processing, OSN's ability to concentrate solutes makes it invaluable in the chemical and pharmaceutical industries. A variety of organic solvents can be recovered through the OSN process, including alcohols like methanol, ethanol, isopropanol, and n‐butanol, as well as aromatic hydrocarbons like toluene, xylene, and benzene, promoting energy reuse. This membrane‐based process presents multiple benefits compared to traditional separation methods, such as enhanced energy efficiency, mild operating conditions, and solvent recovery and recycling potential. However, the chemical durability of the membrane is a critical factor, especially when operating in organic solvents. Among the various techniques for producing COF membranes, imine‐based, hydrazine‐linked, and ketoenamine‐linked COFs are known for their relative stability and suitability for OSN applications.

**Figure 15:**
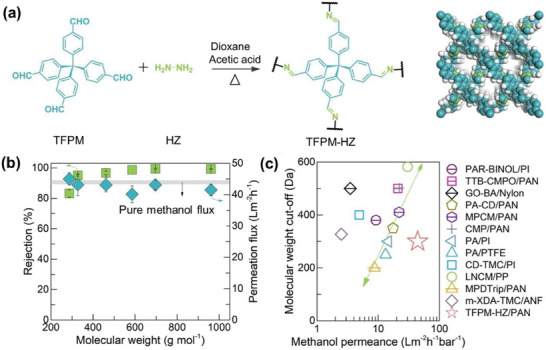
a) Schematic diagram for the synthesis and molecular structure of TFPM‐HZ. b) Methanol flux and rejection rates observed during the filtration of solutions containing various molecules. c) Comparison of OSN performance from this study with previous research. Reproduced with permission.^[^ ^29^ ^]^ Copyright 2022, Wiley‐VCH.

In 2022, Wang and co‐workers synthesized a novel 3D COF, TFPM‐HZ, by condensation of tetrakis(4‐formylphenyl)‐methane (TFPM) and hydrazine hydrate (HZ), featuring sub‐nanometer channels and an interpenetrated pore network.^[^ ^29^ ^]^ To control the crystallization process of TFPM‐HZ, nanoporous PAN supports were employed. These supports facilitated the formation of a dense, uniform 3D COF separation layer on the support surface through controlled interfacial polymerization (**Figure** **15**a). Extensive characterization confirmed the highly crystalline nature and uniform sub‐nanometer pores of the synthesized TFPM‐HZ layer. The TFPM‐HZ/PAN composite membrane displayed remarkable performance in OSN (Figure 15b,c). These membranes demonstrated remarkably high methanol permeance (44 L m^−2^ h^−1^ bar^−1^) while maintaining sharp molecular sieving ability (>95% rejection). Notably, the TFPM‐HZ/PAN membrane demonstrated excellent stability when exposed to concentrated pharmaceutical feeds, enabling prolonged operation for over 1000 h. This study effectively demonstrated the viability of the TFPM‐HZ/PAN membrane for purifying active pharmaceutical ingredients from organic solvents. It emphasizes the significant promise of carefully designed 3D COFs in developing high‐performance OSN membranes with tailored structures suitable for industrial applications. The robust crystalline skeletons and interconnected sub‐nanometer channels of 3D COFs enable superior permeance and molecular sieving while mitigating the swelling and aging issues commonly associated with traditional membranes.

**Figure 16:**
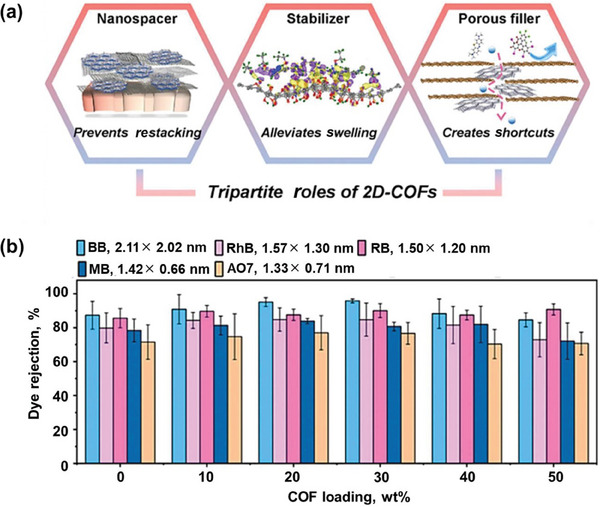
a) The threefold function of 2D‐COF in membranes for OSN based on graphene. b) Rates of rejection for different organic dyes in methanol with varying amounts of COF‐LZU1. Reproduced with permission.^[^ ^139^ ^]^ Copyright 2021, Elsevier.

In the field of OSN, GO is frequently incorporated to enhance membrane performance.^[^ ^137^, ^138^ ^]^ However, the specific role of COF in the composite membrane formed with GO has yet to be thoroughly investigated. In 2021, Zhou, Goh, Chen, and co‐workers developed a COF‐LZU1@rGO membrane by combining COF‐LZU1 with reduced graphene oxide (rGO).^[^ ^139^ ^]^ The optimal COF‐LZU1@rGO demonstrated a 162% increase in methanol permeance compared to the unmodified rGO while preserving significant rejection rates for organic dye solutes. The investigation through experimental characterization and density functional theory computations demonstrated that solvation prompts a charge redistribution at the interface between COF‐LZU1 and rGO. This phenomenon resulted in an attractive force that helped maintain the interlayer distance and reduced excessive swelling of rGO in solvent environments. This work demonstrates that the 2D COFs play three critical roles: acting as nanospacers to prevent restacking of rGO sheets, stabilizers to alleviate rGO swelling in organic solvents, and as porous fillers to create shorter transport pathways for enhanced solvent permeance (**Figure** **16**a,b). Furthermore, the well‐defined in‐plane pores within the COFs facilitated shorter transport routes, allowing for faster permeance of solvent molecules.

**Figure 17:**
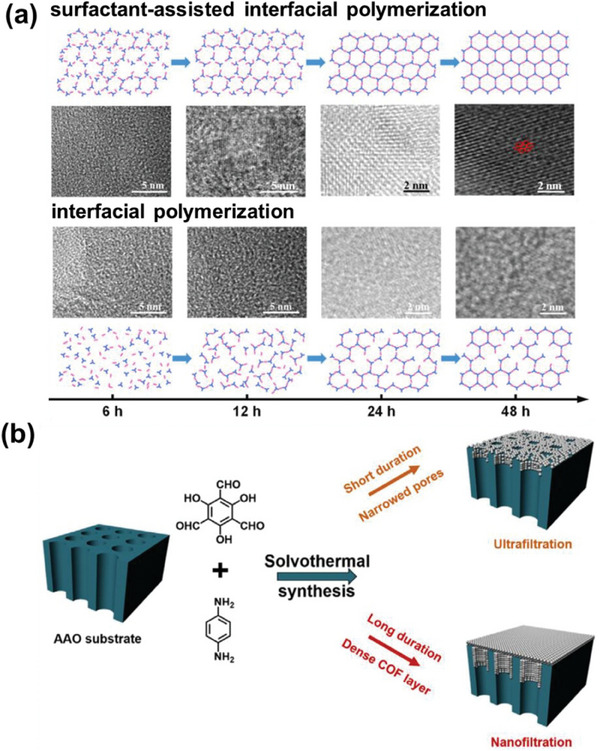
a) HRTEM images of TpPa membranes produced through interfacial polymerization and surfactant‐assisted interfacial polymerization techniques with varying durations of polymerization, alongside schematic representations of the respective processes. Reproduced with permission.^[^ ^96^ ^]^ Copyright 2024, Elsevier. b) Schematic illustration of the synthesis of TpPa‐AAO membranes intended for ultrafiltration and nanofiltration applications. Reproduced with permission.^[^ ^142^ ^]^ Copyright 2019, Elsevier.

With the emergence of COF‐based OSN membranes, techniques such as interfacial polymerization, layer‐by‐layer assembly, and in situ growth have been suggested to improve their effectiveness. In 2024, Wu, Li, and co‐workers employed a surfactant‐assisted strategy for interfacial polymerization to produce TpPa COF membranes with high crystallinity.^[^ ^96^ ^]^ The self‐assembled amphiphilic surfactant chains at the oil‐water interface facilitated the pre‐assembly and organized arrangement of monomers, promoting their complete topological polymerization into a highly crystalline framework (**Figure** **17**a). Due to their well‐defined pore architecture, these surfactant‐assisted interfacial polymerization TpPa COF membranes exhibited enhanced nanofiltration efficiency compared to membranes prepared by conventional interfacial polymerization. This was evidenced by a 77.0% increase in acetonitrile permeance (366.9 L m^−2^ h^−1^ bar^−1^) and an improved rejection rate of 95.7% for reactive black dye (2.0 nm). Notably, the robust crystalline framework demonstrated exceptional solvent resistance, high compressive strength, and operational durability, ensuring consistent performance for over 100 h. In 2023, Su and co‐workers successfully performed interfacial polymerization by dissolving p‐phenylenediamine and 1,3,5‐triformylphloroglucinol in aqueous m‐phenylenediamine solutions and organic trimesoyl chloride solutions, followed by heat treatment.^[^ ^140^ ^]^ The resulting nanocomposite OSN membrane was fabricated via a one‐step in‐situ co‐polymerization process. The optimized membrane, TFN‐100‐75, showed an ethanol permeance of 65.7 L m^−2^ h^−1^ MPa^−1^ and achieved a rejection rate of 99.0% for Rhodamine B (RDB, 479 Da) while employing exceptionally low concentrations of reactive monomers and m‐phenylenediamine. Jiang, Wu, and co‐workers developed an oriented ionic COF (TpPa‐SO_3_H) membrane using a layer‐by‐layer stacking approach, employing high‐aspect‐ratio crystalline TpPa nanosheets functionalized with sulfonate groups.^[^ ^141^ ^]^ Characterization revealed well‐organized channels in the oriented ionic TpPa‐SO_3_H membrane that were vertically aligned with the membrane surface and enriched with negative charges from the sulfonate functional groups. In the context of OSN, the oriented ionic TpPa‐SO_3_H membrane demonstrated superior performance in solvent permeance and molecular sieving selectivity. It successfully rejected over 99% of the negatively charged dye Evans blue while sustaining an ethanol permeance of 63 L m^−2^ h^−1^ bar^−1^. Significantly, the membrane exhibited significantly higher rejection for negatively charged dyes than positively charged dyes of similar size, with up to a 3‐fold difference. This demonstrated the oriented ionic TpPa‐SO_3_H membrane's ability to leverage electrostatic repulsion effects, in addition to size exclusion, to enhance selectivity.

Pressure‐driven membrane separation methods, such as nanofiltration, ultrafiltration, and microfiltration, have garnered significant attention in various industrial applications, including water treatment,^[^ ^143^, ^144^, ^145^ ^]^ food processing,^[^ ^146^, ^147^ ^]^ and biotechnology.^[^ ^148^, ^149^ ^]^ These processes primarily differ in their ability to reject solutes based on molecular weight cutoff or pore size. However, transitions between nanofiltration and ultrafiltration can be achieved through special modifications. In 2019, Wang and co‐workers demonstrated that by altering the synthesis duration, the pore size of the COF substrate (AAO) could be controlled, allowing for a transition between ultrafiltration and nanofiltration.^[^ ^142^ ^]^ This approach effectively enhanced the membrane's selectivity. The microporous TpPa COF synthesized in the research reduced the pore size of the AAO substrate while introducing additional nanochannels for water transport, resulting in a permeance approximately 2–9 times higher than that of other ultrafiltration membranes with comparable rejection rates (Figure 17b).

**Table** **1** provides a comprehensive comparison of the performance of four types of membranes—COF, MOF, amorphous polymer, and cross‐linked polymer—in three separation processes: gas separation, oil/water separation, and organic solvent nanofiltration. Due to the strong covalent bonds present in the COF membrane, these membranes can maintain their integrity in harsh chemical environments and at elevated temperatures. This characteristic renders COF membranes more stable than MOF membranes, which depend on coordination bonds that are more susceptible to degradation under acidic, alkaline, or high‐temperature conditions. Furthermore, COF membranes exhibit a high degree of structural tenability. By systematically designing organic monomers, their pore sizes can be precisely controlled and functionalized to enable selective molecular recognition and separation. This property enables COF membranes to surpass the pore size limitations inherent in MOFs, where metal‐organic coordination geometries restrict flexibility, and tailoring pore structures in polymer membranes poses additional challenges. With their well‐defined and tunable pore channels, COFs offer high selectivity in molecular separations, making them particularly suitable for applications including gas separation, oil/water separation, and organic solvent nanofiltration. Additionally, COF membranes typically exhibit high permeance due to their inherent porosity and ordered channels, which promote efficient material transport. Despite these advantages, the large‐scale production and fabrication of COF membranes remain challenging. Synthesizing defect‐free COF structures over large areas and ensuring consistent mechanical stability in membrane applications are significant hurdles. Another challenge facing COF membranes is their mechanical properties. Many COFs are more brittle than polymers, limiting their flexibility and making them less suitable for applications requiring mechanical strength.

**Table 1:** The separation performance of COF membranes, MOF membranes, amorphous polymer, and cross‐linked polymer.

Separation type	Membrane name	Membrane material	Separation efficiency/Selectivity	Permeance	Ref.
Gas separation	TAPB‐BTCA‐COF/GO	COF	15.6	366 Barrer	[123]
	LA‐*α*‐CD‐in‐TpPa‐1	COF	30.6	3000 GPU	[98]
	COF‐316	COF	50.0	500 GPU	[150]
	N‐COF	COF	13.8	4319 GPU	[122]
	COF‐300	COF	11.0	5160 GPU	
	ZIF‐67‐in‐TpPa‐1	COF/MOF^a)^	34.9	3800 GPU	[127]
	TpPa‐2 SSSN	COF	22.0	328 GPU	[89]
	TpTGCl@TpPa‐SO_3_H/COF‐LZU1	COF	26.0	2163 GPU	[124]
	COF@XLPEO	COF	61.4	803.9 Barrer	[151]
	Pebax‐PEG@COF	COF	33.0	944 Barrer	[152]
	TpEBr@TpPa‐SO_3_Na iCON	COF	22.6	2566 GPU	[125]
	vertical COF‐LZU1	COF	31.6	3600 GPU	[126]
	DhaTGCl iCOF	COF	49	316 GPU	[20]
	H_2_P‐DHPh COF−UiO‐ 66	COF/MOF^a)^	32.9	108 341 Barrer	[128]
	TB/ZIF‐8@PDA	MOF	37.7	664.1 Barrer	[153]
	PI/IPD@ZIF‐8	MOF	15.1	8000 Barrer	[154]
	ZIF‐8/polyether Sulfone	MOF	35.1	3.3×10^−7^ mol m^−2^ s^−1^ pa^−1^	[155]
	PIM1‐Zn_2_(bim)_4_	MOF	10.5	1358 Barrer	[156]
	PIM1‐MOF74	MOF	15.0	1682 Barrer	
	PIM1‐MIL53	MOF	18.3	1027 Barrer	
	PIM1‐TIFSIX3	MOF	19.4	1010 Barrer	
	PAR‐TTSBI	Amorphous polymer	45.0	2115 GPU	[157]
	6FDA‐DAM_0.7_‐TFMB_0.1_‐DCB_0.2_‐400	Cross‐linked Polymer	36.0	198 Barrer	[158]
	PI‐Im‐COOH‐450‐2	Cross‐linked Polymer	38.1	685.1 Barrer	[159]
	6FDA‐DAM polyimide	Cross‐linked Polymer	25.8	483.6 Barrer	[160]
Oil/Water separation	TPB‐DMTP‐COF/PAN	COF	98.0%	8133 L m^−2^ h^−1^	[131]
	TpHZ COF	COF	99%	38 842 L m^−2^ h^−1^ bar^−1^	[90]
	COF‐Q‐CF_3_	COF	99.6%	2.2 × 10^4^ L m^−2^ h^−1^	[161]
	SH‐C4‐COF	COF	99%	‐	[162]
	PA@COF	COF	99.7%	4583 L m^−2^ h^−1^	[132]
	Wood/COF	COF	99%	‐	[134]
	TFB‐BD(Me)_2_ COF	COF	99.7%	18 000 L m^−2^ h^−1^	[135]
	COF/GO_50_	COF	98.3%	26 960 L m^−2^ h^−1^ bar^−1^	[133]
	MOF‐5@PDA	MOF	78.94%	209.02 L m^−2^ h^−1^	[163]
	PVDF@MOF‐303	MOF	99.2%	4200 L m^−2^ h^−1^ bar^−1^	[164]
	Cu‐CAT‐1@CM	MOF	99.9%	329 kL m^−2^ h^−1^	[165]
	MOF‐5@CFM	MOF	99.9%	8861 L m^−2^ h^−1^	[166]
	ZIF‐9‐III@PVDF	MOF	99.8%	‐	[167]
	PPy‐NaLS	Amorphous polymer	99.93%	70.14 L m^−2^ h^−1^	[168]
	PPy‐AOT	Amorphous polymer	90%	‐	[169]
	M–PD/PT/2:2	Amorphous polymer	99.2%	1121.1 L m^−2^ h^−1^	[170]
	FG	Amorphous polymer	99.0%	2086 L m^−2^ h^−1^	[171]
Organic Solvent Nanofiltration	SAIP‐TpPa	COF	95.7%	366.9 L m^−2^ h^−1^ bar^−1^	[96]
	TFN‐100‐75	COF	99.0%	184.3 L m^−2^ h^−1^ MPa^−1^	[140]
	*oi*‐COM	COF	99.0%	63 L m^−2^ h^−1^ bar^−1^	[141]
	PES‐COF‐0.8 wt% GO	COF	96.0%	67.5 L m^−2^ h^−1^ bar^−1^	[137]
	TFG‐EDA	COF	98.0%	531 L m^−2^ h^−1^ bar^−1^	[172]
	TAPB‐TPOC_1_	COF	90.0%	78.2 L m^−2^ h^−1^ bar^−1^	[121]
	TFPM‐HZ/PAN	COF	95.0%	44 L m^−2^ h^−1^ bar^−1^	[29]
	TpPa‐2 COF membrane	COF	100.0%	43.89 L m^−2^ h^−1^ bar^−1^	[173]
	COF‐LZU1/rGO	COF	96.0%	135 L m^−2^ h^−1^ bar^−1^	[139]
	PCN‐250 MOF	MOF	93.7%	337.9 L m^−2^ h^−1^ bar^−1^	[174]
	HKUST‐1	MOF	98.8%	9.59 L m^−2^ h^−1^ bar^−1^	[175]
	*t*‐Cu‐TCPP/P84 MMM	MOF	95.7%	2.82 L m^−2^ h^−1^ bar^−1^	[176]
	CuBDC/CNTs	MOF	95.0%	1260 L m^−2^ h^−1^ bar^−1^	[177]
	COP	Amorphous polymer	91.0%	10.9 L m^−2^ h^−1^ bar^−1^	[178]
	TPB‐F‐BOP	Amorphous polymer	1402 g/mol	37 L m^−2^ h^−1^ bar^−1^	[179]
	TTB‐CMPO	Amorphous polymer	500 g/mol	21 L m^−2^ h^−1^ bar^−1^	[180]
	TFN‐2	Amorphous polymer	328 Da	13.7 L m^−2^ h^−1^ bar^−1^	[30]
	IA membrane	Cross‐linked polymer	11.5	0.2 L m^−2^ h^−1^ bar^−1^	[181]

^a)^ Hybrid materials of COF and MOF.

## 4 Conclusion

The exceptional porosity, extensive surface area, and tunable pore sizes of COFs make them highly promising for selective separation applications. By carefully selecting and modifying organic linkers and through post‐synthesis modifications, COF membranes can be engineered to exhibit desirable properties, including high mechanical strength, enhanced chemical stability, and switchable wettability. These characteristics position COFs as ideal materials for membrane design and separation technologies. To implement COFs in separation membranes, methods such as interfacial polymerization, layer‐by‐layer assembly, and in situ growth are commonly employed. These membranes demonstrate outstanding performance in applications such as gas separation, oil/water separation, and organic solvent nanofiltration, highlighting the significant potential of COFs in membranes. Notably, by controlling membrane thickness, a balance between permeance and selectivity can be achieved, allowing for high selectivity while maintaining high permeance. Different preparation methods result in varying membrane thicknesses and performance characteristics, making them suitable for different separation applications. While COF membranes have made substantial strides in separation technologies, several challenges hinder their widespread practical application. These challenges include:

- Scaling up COF Membrane Production: The precise conditions required for synthesis, such as temperature, pressure, and stoichiometry, make scaling up from laboratory to industrial production a significant hurdle. Developing more reliable and scalable methods is crucial for overcoming this obstacle. To achieve large‐scale production, alternative methods beyond interfacial polymerization and in situ growth are necessary. While layer‐by‐layer assembly offers potential, further advancements are needed to scale up production. Additionally, incorporating COFs into mixed matrix membranes, leveraging existing industrial facilities, presents a promising approach for industrial‐scale production.
- Stability Under Harsh Conditions: The stability of COF membranes under prolonged exposure to harsh industrial conditions, including variations in temperature, pH, and corrosive solvents, is a major concern. Enhancing the chemical stability of COF membranes is essential for their suitability in practical industrial applications. To enhance the stability of the COF membrane, it is essential to focus on designing stable covalent linkages or modifying the membrane through surface functionalization.
- Mechanical Strength: The uniformity and defect‐free structure of COF membranes may be compromised under the high operating pressures and flow rates typical of industrial processes. Improving the mechanical strength of COF membranes is crucial for advancing materials science in this field. To enhance the mechanical strength of COF membranes, future research should focus on strategies such as strengthening interlayer interactions, optimizing structural design, and developing composite materials. These approaches have the potential to significantly improve the mechanical integrity of COF membranes, expanding their durability and applicability in demanding environments.
- Environmental Impact: The synthesis of COF membranes often involves the use of organic solvents and energy‐intensive processes. Developing greener and more sustainable preparation methods to minimize environmental impact and resource consumption is a significant challenge for the future development of COF membranes. To mitigate the environmental impact of COF membrane manufacturing, future strategies should prioritize the development of green synthesis routes, such as preparation at room temperature or in an aqueous solution. These methods aim to minimize the use of organic solvents and reduce energy consumption, thereby promoting the production and application of COF membranes.

Despite these challenges, the unique features of COF‐based separation membranes, including their high porosity, tunable pore size, and richly functionalized surfaces, offer significant advantages for efficient separation processes. This review aims to assist researchers in enhancing membrane preparation techniques and broadening the range of COF membrane applications.

## Conflict of Interest

The authors declare no conflict of interest.
